# A comparative study on effect of news sentiment on stock price prediction with deep learning architecture 

**DOI:** 10.1371/journal.pone.0284695

**Published:** 2023-04-25

**Authors:** Keshab Raj Dahal, Nawa Raj Pokhrel, Santosh Gaire, Sharad Mahatara, Rajendra P. Joshi, Ankrit Gupta, Huta R. Banjade, Jeorge Joshi

**Affiliations:** 1 Department of Statistics, Truman State University, Kirksville, MO, United States of America; 2 Department of Physics and Computer Science, Xavier University of Louisiana, New Orleans, LA, United States of America; 3 Department of Physics, The Catholic University of America, Washington, DC, United States of America; 4 Department of Physics, New Mexico State University, Las Cruces, NM, United States of America; 5 TQuT Inc., Rockford, MI, United States of America; 6 Department of Computer Science, Central Michigan University, Mount Pleasant, MI, United States of America; 7 Department of Physics, Virginia Commonwealth University, Richmond, VA, United States of America; 8 Kathmandu Engineering College, Tribhuvan University, Kathmandu, Nepal; Huazhong University of Science and Technology, CHINA

## Abstract

The accelerated progress in artificial intelligence encourages sophisticated deep learning methods in predicting stock prices. In the meantime, easy accessibility of the stock market in the palm of one’s hand has made its behavior more fuzzy, volatile, and complex than ever. The world is looking at an accurate and reliable model that uses text and numerical data which better represents the market’s highly volatile and non-linear behavior in a broader spectrum. A research gap exists in accurately predicting a target stock’s closing price utilizing the combined numerical and text data. This study uses long short-term memory (LSTM) and gated recurrent unit (GRU) to predict the stock price using stock features alone and incorporating financial news data in conjunction with stock features. The comparative study carried out under identical conditions dispassionately evaluates the importance of incorporating financial news in stock price prediction. Our experiment concludes that incorporating financial news data produces better prediction accuracy than using the stock fundamental features alone. The performances of the model architecture are compared using the standard assessment metrics —Root Mean Square Error (RMSE), Mean Absolute Percentage Error (MAPE), and Correlation Coefficient (R). Furthermore, statistical tests are conducted to further verify the models’ robustness and reliability.

## 1 Introduction

The stock market allows us to buy and sell units of ownership in a company called stocks, and one can own some of those profits when a company’s profit goes up and vice versa. The evolution of the stock market is intriguing, from handwritten stock trades in coffee shops to today’s digital platform, where one can have access to the entire world’s stock market in the palm of one’s hand. Thus, it boosts economic growth by encouraging competition and innovation in different sectors including business, education, and labor market [1, 2]. However, purchasing the right stock at the right time is one of the most challenging jobs. Several reasons and circumstances behind the scene affect stock price due to its highly nonlinear, volatile, fuzzy, noisy, non-parametric, deterministic chaotic behavior by default [3–5]. The stock price prediction model helps us minimize the level of uncertainty for better investment and trading decisions if and only if the well-balanced combination of features that affect the stock price are properly utilized.

There have been various schools of thought used in stock price prediction. Traditionally, the first wave believes in the efficient market hypothesis theory [6]. It urges historical stock data has much influence in predicting the future price. Conversely, the concept of random walk [7] believes that a particular stock’s price is already reflected in its current price. Then any change in the stock price would reflect the release of new information or random noise. The second wave focuses on statistical modeling, where the central focus is predicting future prices based on the relationship between past and present data [8–13]. Almost all of these models associate the linear relationship between the given variables, but stock market data are often nonlinear. The third and most potent wave came into existence due to technological advancement, high computational power, and rule-based artificial intelligence algorithm growth. These models primarily help to capture the nonlinear behavior of the stock market data.

With very few exceptions, most models developed under any wave utilize structured numerical data to predict stock prices. These data alone are not sufficient to examine stock returns, forecasting daily and weekly market patterns [14, 15]. In the recent decade, the stock market movement has been influenced by public or private information shared via different digital media platforms such as Facebook posts, tweets on Twitter, or financial gossip on multiple platforms. All these activities further increase the stock market volatility as the information is more inclined toward the psychological thought processes of human beings. When the discussion revolves around human beings and human sentiment, the situation is intricate and complex. A case in point is on January 5, 2017, President-elect Donald Trump tweeted to impose a hefty tax on Toyota Motor if builds its Corolla cars for the U.S. market at a plant in Mexico. This tweet had a substantial impact on Toyota stock as its price dipped and the volume spiked [16, 17]. Similarly, stocks fell on August 1, 2019, right after president Donald Trump posted a series of tweets about the 10% charge that would be imposed on $300 billion worth of Chinese goods. The Dow Jones Industrial Average closed 98.41 points lower at 26,485.01 after plunging 334.20 points earlier in the day. The S&P 500 lost 0.7% to end the day at 2,932.05. (https://www.cnbc.com). Furthermore, the famous tweet by Elon Musk on accepting Bitcoin as a payment method for Tesla cars. On the same day stock price of Bitcoin increased by 5.2% on 24 march 2021. The stated evidence speaks volumes of information about the importance and influence of market sentiment in stock price prediction.

Many research articles have been published so far using variations of deep learning models, and varying levels of claims have been seen concerning the models’ accuracy and robustness [18–23]. The most popular deep learning architecture are Long Short Term Memory (LSTM), Gated Recurrent Unit (GRU), Convolution Neural Network, and their respective hybridization techniques [18, 24–33]. Every new publication speaks with pride about the accuracy and model’s robustness. Their implementation strategies, working framework, series of assumptions, number of features, and data sources differ from one to another. Thus, it is impractical to make an unbiased comparison between previously published articles that use the same model to predict the stock price. Furthermore, the literature lacks a comparative analysis of stock price prediction with or without incorporating unstructured data on regular stock market data.

This study compares LSTM and GRU models for stock price prediction under the standard framework using identical conditions. It helps to objectively assess the statistical significance of including or excluding financial news sentiment in stock price prediction. Financial news of the developed countries is captured in multiple media outlets. The same news is published on various platforms with a micro analysis of the subject area from multiple avenues. Due to the heterogeneity and diversity of assessing the news sentiment for stock market data over a certain period, it is rational to select the data from developed countries to support the purpose of the study.

**Fig 1:**
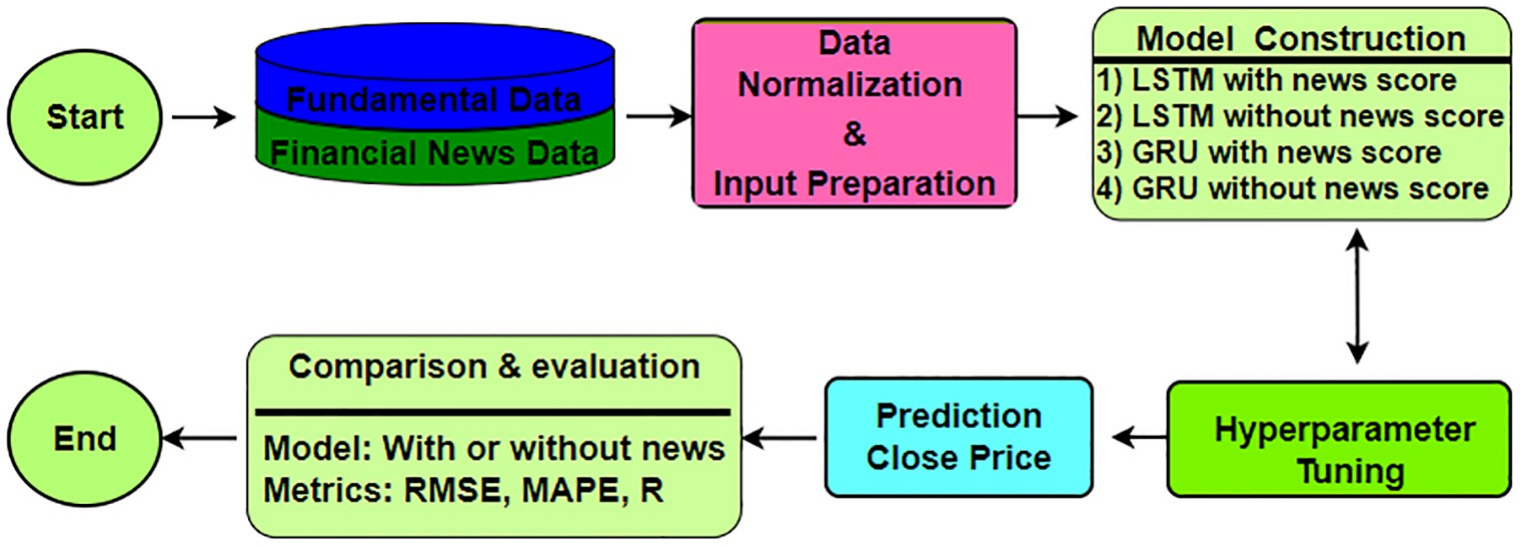
Overall schematic diagram of the proposed research framework.

Our complete vision to achieve the stated goal can be conceptualized from the broader spectrum via the schematic diagram in Fig 1. The proposed study uses fundamental and financial news data to build the model. The concatenated data is normalized using the min-max technique. LSTM and GRU models are developed with or without incorporating financial news data. Once the hyperparameters are tuned, the final model predicts the closing price of the stock market index. The final model helps to determine whether or not the financial news influences the stock price prediction. At the end, the quality and robustness of the proposed model are assessed through RMSE, MAPE, and R scores.

The main contributions of this study include (a) Answering the central question: under identical conditions, which LSTM or GRU model would be the best choice for stock price prediction? (b) Confirming the statistical causal inference of news sentiment on the stock price prediction. (c) Determining the significance of incorporating the news sentiment in stock price prediction concerning if only fundamental stock attributes are utilized. (d) Conducting a series of statistical hypotheses to validate the experiment’s robustness and reliability.

The rest of the paper is organized as follows. In Section 3, we explain the method of preprocessing the data before using it in the ML models. Section 4 explains the modeling approach. In section 4.3, we discuss the brief model performance metrics. Section 5 explains the experimental settings and results. Finally, section six presents the conclusion, followed by a list of references.

## 2 Related work

The study conducted by Adebiyi et al. [34] evaluated the performance of an Artificial Neural Network (ANN) against an Autoregressive Integrated Moving Average (ARIMA) model using historical stock data of Dell Inc. on the New York Stock Exchange (NYSE). The research concluded that the ANN model performed slightly better than the ARIMA model and noted that incorporating macroeconomic and technical indicators could further improve the results.

In a 2019 study, Karmiani and colleagues [35] compared the performance of LSTM, Backpropagation, SVM, and Kalman filter for stock price prediction. They used historic data from Yahoo Finance for nine companies (Apple, Acer, Amazon, Google, HP, IBM, Intel, Microsoft, and Sony) and found that LSTM had the highest prediction accuracy and lowest variance among the models tested.

Chen et al. (2015) [36] used LSTM to predict stock prices in the China stock market, using historical data from the Shanghai and Shenzhen stock markets obtained from Yahoo finance as input features. They reported an accuracy of 27.2% and suggested that incorporating other features such as macroeconomic data and technical indicators would improve the model’s performance.

In the study by Roondiwala et al. [37], LSTM was utilized to predict future stock prices of NIFTY50. Historical data, including high, low, open, and close prices, was obtained from the National Stock Exchange and used as input features. The RMSprop optimizer was employed with 500 epochs, resulting in a testing RMSE score of 0.00859. However, it is possible that normalized data were used to calculate the RMSE, rather than actual data. Additionally, the model’s performance could have been improved by incorporating other factors, such as financial sentiments, that have a direct impact on stock prices.

In the study by Yu and Yan [38], data for six stock indices from various market environments were used, including the S&P 500, DJIA, N 225, HSI, CSI 300, and ChiNext index. In the first stage, the authors applied phase-space reconstruction (PSR), de-noising, and normalization to the data to improve the performance of the model. Four standard machine learning algorithms were compared: LSTM, MLP, SVR, and ARIMA. The results showed that the LSTM had the highest prediction accuracy among the algorithms compared.

In the work of Gao et al. the group applied four neural networks named Multilayer Perceptron (MLP), Long Short Term Memory (LSTM), Convolutional Neural Network (CNN) and one attention-based neural network —Uncertainty-aware Attention (UA)—to test the performance on predicting three stock market price: the SP500 index (most developed market), CSI300 index (less developed market) and Nikkei225 index (developing market) [39]. The results show that UA has the best performance among the alternative models. Furthermore, all models have better accuracy in the developed financial market than in developing ones.

In their study, Shahi et al. (2020) [40] investigated if incorporating financial news sentiments could improve the performance of stock price prediction using LSTM and GRU models. They used historical data from the Agricultural Development Bank Limited (ADBL) of Nepal and financial news headlines from ShareSansar Nepal, from 20 March 2011 to 14 November 2019. The results showed that the performance of both LSTM and GRU models was significantly improved by including financial news sentiments as input features.

Kara et al.in 2011 [41] compared two classifiers —Artificial neural networks (ANN) and support vector machines (SVM) —to predict the direction of movement in the daily Istanbul Stock Exchange (ISE) National 100 Index. The data from January 2, 1997 to December 3, 2007 were taken. Then technical indicators: Simple 10-day moving average, Weighted 10-day moving average, Momentum, Stochastic K%, Stochastic D%, Relative Strength Index (RSI), moving average convergence divergence (MACD), Larry William’s R%, Accumulation/Distribution Oscillator, and Commodity Channel Index were selected as input variables. Experimental results showed that the average performance of ANN model (75.74%) was found significantly better than that of SVM model (71.52%).

In the work of Schoneburg, E. [42], the author analyzed the possibility of predicting stock prices on a short-term, day-to-day basis with the help of neural networks —Perceptron, Adaline, Madaline, and Backpropagation —by studying three important German stocks: BASF, COMMERZBANK, MERCEDES. The author achieved an accuracy of up to 90% with a back propagation network. Moreover, he expresses that the selection of more suitable inputs for the network could improve the performance of the model.

K. Kohara et al. [43] used neural networks (NNs) for the prediction of the daily closing price of Tokyo stock price index (TOPIX) whether incorporating Event-knowledge (the daily headlines of the Japanese newspaper) could produce a better performance. The five inputs —Close: closing price of TOPIX, Exchange: the dollar-to-yen exchange rate (yen/dollar), Interest: an interest rate, Oil: the price of crude oil, and NY: New York Dow-Jones average of the closing prices of 30 industrial stocks —with and without Event-knowledge, feed to the NNs. The result shows that the performance of NNs is improved significantly by incorporating Event-knowledge.

Adebiyi A. A. et al. [44] used feed forward multilayer perceptron neural network with backpropagation whether incorporating fundamental analysis variables could produce a better performance than technical analysis variables only. The published stock data obtained from the Internet were used. The empirical results show that the performance of the model improved significantly by incorporating fundamental analysis variables for daily stock price prediction.

Selvin S. et al. [45] used Recurrent Neural Networks (RNN), Long Short Term Memory(LSTM), and Convolutional Neural Network (CNN) for short term stock price prediction using a sliding window approach. The window size was 100 minutes with 90 minute’s information and prediction was made for the rest of the 10 minutes. Two companies from IT sector and one company from the Pharma sector of NIFTY were taken for the study. For their proposed methodology, CNN is identified as the best model.

During the COVID-19 pandemic in 2021, Binrong Wu. et al. [20] utilized the following machine learning models to forecast oil price, oil production, oil consumption, and oil inventory: CNN (Convolutional neural network), BPNN (Backpropagation neural networks), SVM (Support vector machines), LSTM (Long short-term memory), and RNN (Recurrent neural network). Their empirical findings suggest that information gleaned from social media platforms makes a major contribution to the process of forecasting oil prices, production levels, and consumption rates.

## 3 Data preparation and alignment

Broadly speaking, this study uses two types of datasets —SP&500 stock market index and financial news data. The overall description of the dataset is presented in Table 1. Stock market data consist of stock data for a popular US stock market index; S&P500, between June 9, 2008, to November 5, 2021 accessed from Yahoo Finance [46]. It consists of the open, close, maximum, and minimum stock price as well as the shares traded (volume) on a particular day.

**Table 1:** Overall description of the datasets.

Dataset	Features	Source	Date	Frequency
Historical Stock Data	Open, Close, High, Low, Volume	Yahoo Reddit World News Channel Finviz	06-09-2008 to 11-05-2021 06-09-2008 to 07-01-2017 08-03-2020 to 11-05-2021	Daily
Financial News Data	News Headlines, News Body			At Least 25 news per day

Financial news data scraped from Reddit World News Channel [47] and Finviz [48]. Reddit World News Channel data are extracted from June 9, 2008, to July 1, 2017, from Kaggle [49]. Similarly, from January 1, 2020, to November 5, 2021, data are collected from Finviz. The news scraped from Finviz has many missing values from January 1, 2020, to August 2, 2020, so we retained the news from August 3, 2020, to November 5, 2021, and the rest was discarded. Reddit news data, and Finviz news data are converted into a numerical score using the VADER (Valence Aware Dictionary for Sentiment Reasoning) package.

VADER [50] is a pre-trained model that analyzes people’s opinions, sentiments, evaluations, attitudes, and emotions via computational treatment regarding polarity (positive/negative) and intensity (strength) in text. It relies on an English dictionary that maps lexical features to their semantic orientation as positive or negative [51].

Before inputting financial news obtained through web scraping into VEDAR for sentiment analysis, the data is first preprocessed. This involves removing any unnecessary text found in HTML tags and single or multiple blank spaces and escape sequences. However, the exclamation marks or question marks found in the news headline are not removed as they may add intensity and strength to the news. After preprocessing, the data is fed into VEDAR to determine its corresponding sentiment scores.

**Fig 2:**
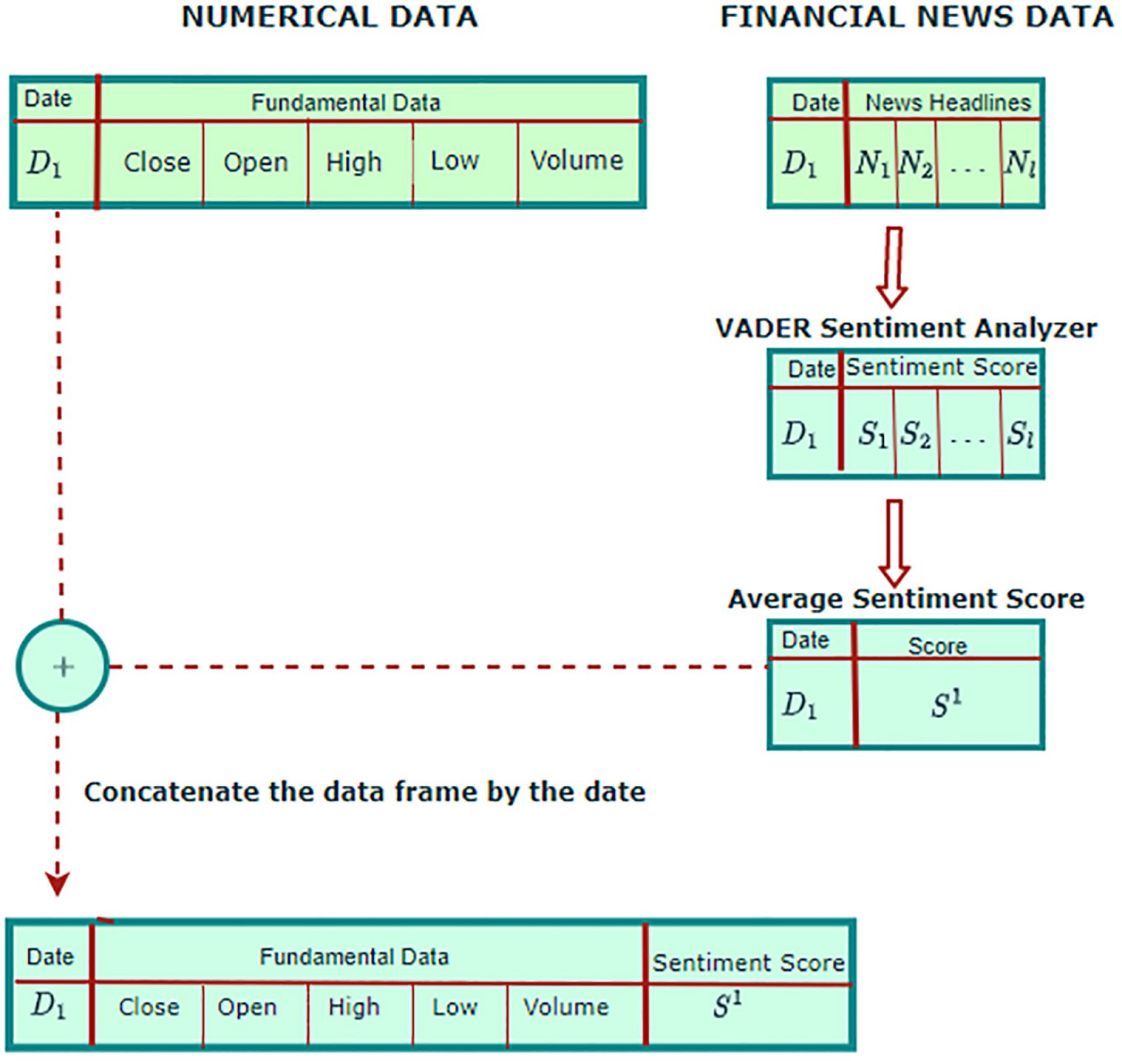
Concatenation of fundamental and financial news data.

Both news data contain at least 25 news published in a single day. So, we computed the average news score for each day. Finally, the sentiment scores for the financial news data are aligned with the stock market data, as shown in Fig 2.

Once the data are concatenated into a single frame based on the date column that exist in both datasets. The descriptive statistics of the combined data is illustrated in Table 2 to gain the initial insight of it. As seen in Table 2, some metrics have large magnitude compared to others. To avoid features with large magnitude dominate the feature with a small magnitude, as partially shown in Table 3, min-max scaling is performed for each metric as defined by Eq 1.

$$\begin{array}{c} z=\frac{x-x_{min}}{x_{max}-x_{min}} \end{array}$$ (1)

where *z*, *x* are scaled and the original input respectively. Similarly, *x*_*min*_ and *x*_*max*_ are the minimum and the maximum values of the input respectively. Prior to feeding the data into the ML models, the scaled 2 dimensional data were converted into 3 dimensions; a number of samples, time step, and the number of features by incorporating the time step. The partial snapshot of the normalized dataset used in this study is presented in Table 4.

**Table 2:** The descriptive statistics of the features.

Statistics	Min	Max	Mean	Std
High	695.27	4718.50	1834.73	942.02
Low	666.79	4681.32	1812.91	936.26
Close	676.53	4697.53	1824.70	639.57
Open	679.28	4699.26	1824.10	938.91
Volume	1.02 × 10^9^	1.14 × 10^10^	4.15 × 10^9^	1.21 × 10^9^
News score	-0.56	0.72	-0.16	0.15

**Table 3:** The snapshot of the actual features used in the model.

Date	Close	Low	Open	High	Volume	News score
2008-06-09	1361.76	1350.62	1360.83	1370.63	4404570000	-0.24
2008-06-10	1358.44	1351.56	1358.98	1366.84	4635070000	-0.30
2008-06-11	1335.49	1335.47	1357.09	1357.09	4779980000	-0.14
2008-06-12	1339.87	1331.29	1335.78	1353.03	4734240000	-0.13
2008-06-13	1360.03	1341.71	1341.81	1360.03	4080420000	-0.25
2021-11-04	4680.06	4662.59	4662.93	4683.00	3332940000	0.12
2021-11-05	4697.53	4681.32	4699.26	4718.50	3491150000	0.14

**Table 4:** The partial snapshot of the normalized dataset.

Date	Close	Low	Open	High	Volume	News score
2008-06-09	0.5327	0.5291	0.5310	0.53056	0.3240	0.4640
2008-06-10	0.5301	0.5300	0.52950	0.5276	0.3461	0.3600
2008-06-11	0.5123	0.5174	0.5280	0.5200	0.3600	0.6290
2008-06-12	0.5157	0.5142	0.5114	0.5167	0.3556	0.6543
2008-06-13	0.5314	0.5222	0.5161	0.5222	0.2930	0.4476
…	…	…	…	…	…	…
2021-11-04	0.9542	0.9538	0.9417	0.9421	0.1782	0.5350
2021-11-05	0.97467	0.9591	0.9534	0.9618	0.1820	0.5386

## 4 Modeling approach

### 4.1 Long short term memory (LSTM)

Long short term memory (LSTM) is an advanced form of recurrent neural network (RNN) known for its robust performance in time series data. It overcomes the main drawbacks of RNN in preserving the information for long-term dependencies due to the problem of vanishing and exploding gradient [52]. LSTM uses memory cells to solve this problem and consists input layer, hidden layer, an output layer, and cell state [53–55].

**Fig 3:**
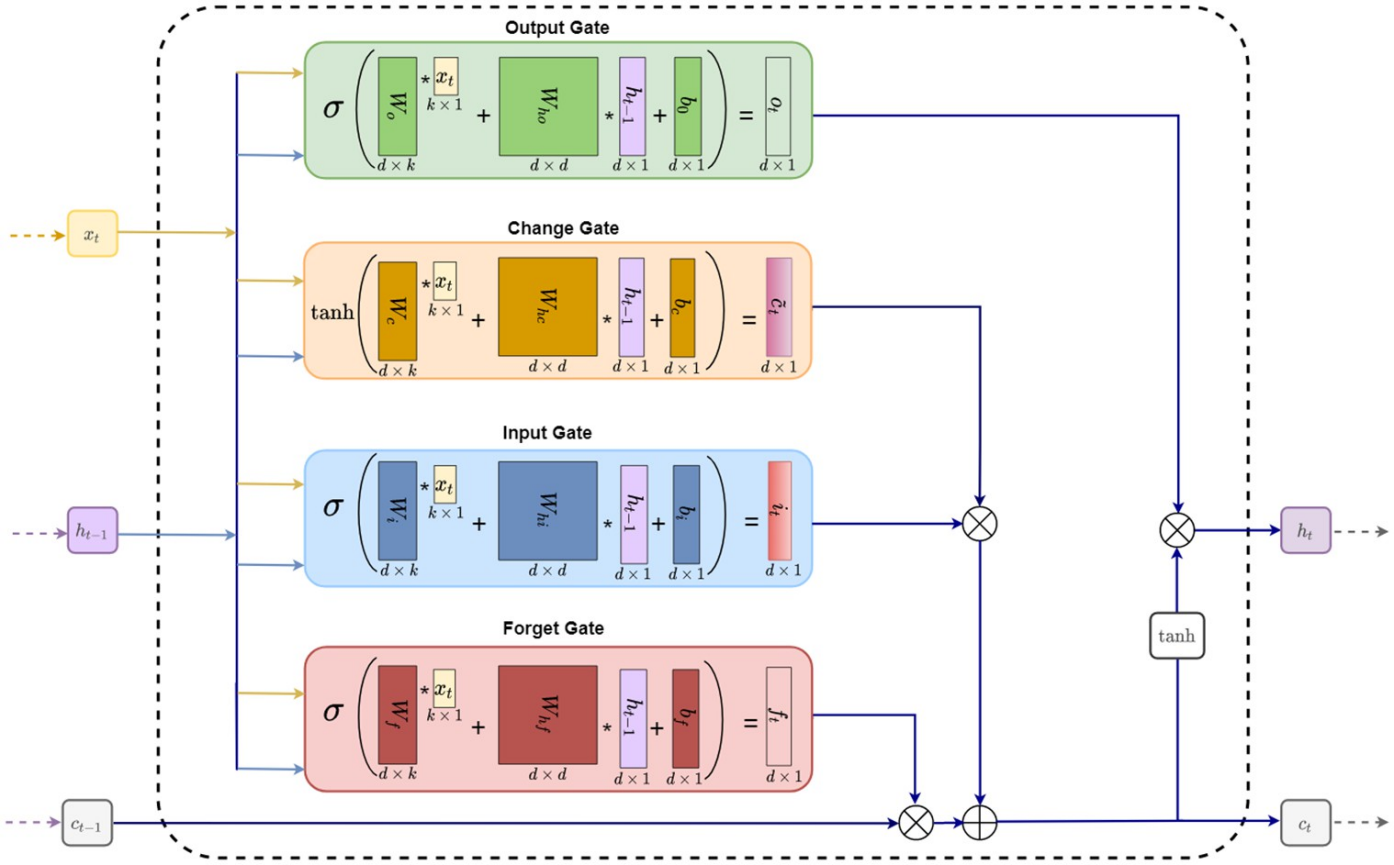
Long short-term memory(LSTM) architecture [56].

Fig 3 illustrates the LSTM architecture. For input *x*_*t*_, the memory cell *c*_*t*_ updates the information using three gates: input gate *i*_*t*_, change gate $\tilde{c_{t}}$, and forget gate *f*_*t*_. The hidden state *h*_*t*_ is updated using output gate *o*_*t*_ and the memory cell *c*_*t*_. These operations of LSTM are governed by the following functions:

$$\begin{array}{cc} & i_{t}=\sigma \left(W_{i}x_{t}+W_{hi}h_{t-1}+b_{i}\right),\\ & f_{t}=\sigma \left(W_{f}x_{t}+W_{hf}h_{t-1}+b_{f}\right),\\ & o_{t}=\sigma \left(W_{o}x_{t}+W_{ho}h_{t-1}+b_{o}\right),\\ & \tilde{c_{t}}=\text{tanh}\left(W_{c}x_{t}+W_{hc}h_{t-1}+b_{c}\right),\\ & c_{t}=f_{t}⊗c_{t-1}+i_{t}⊗\tilde{c_{t}},\\ & h_{t}=o_{t}⊗\text{tanh}\left(c_{t}\right) \end{array}$$

where *σ* and tanh represent the sigmoid and hyperbolic tangent activation functions respectively. The operator ⊗ is the element-wise product, *W*, *W*_*h*_ are the weight matrices, and *b* are bias vectors [56–59].

### 4.2 Gated recurrent unit (GRU)

GRU is a simplified version of the LSTM; first developed by Chung et al. in 2014 [60]. Unlike in LSTM, the short-term state (*h*_*t*_) and long-term state (*c*_*t*_) of LSTM are merged into a single vector *h*_*t*_ in GRU. As opposed to the 3 gates in LSTM, GRU is equipped only with 2 gates: the update gate and the reset gate. The update gate of GRU is equivalent to the forget gate and input gate of LSTM [61]. This gate is responsible for long-term memory. It helps the model to determine how much of the past information needs to be passed along to the future. The reset gate is responsible for short-term memory. It helps the model to decide how much of the past information to forget. Based on empirical evidence, both models; LSTM and GRU have been proven effective on many machine learning tasks [62–66].

**Fig 4:**
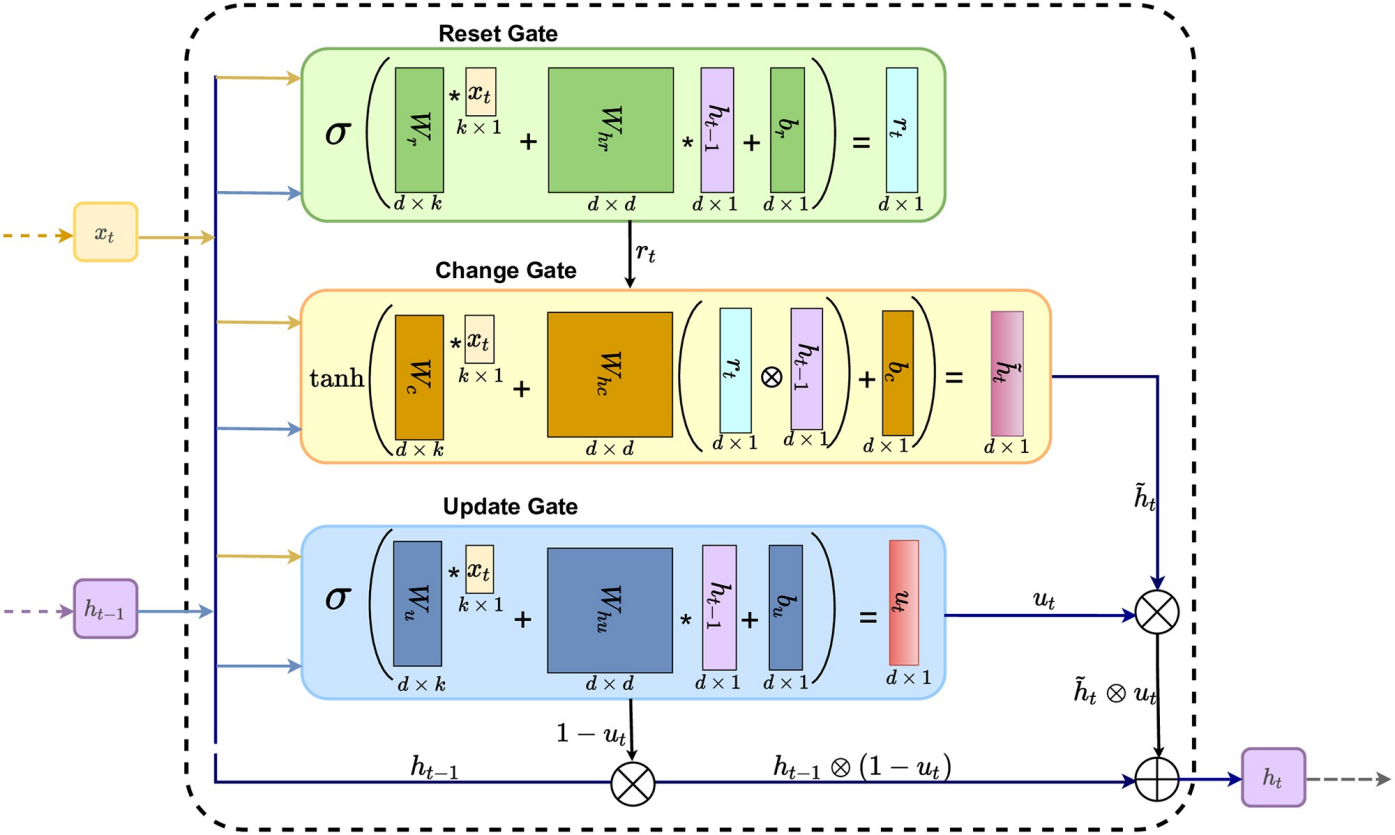
Gated Recurrent Unit (GRU) architecture [66].

Fig 4 illustrates the GRU architecture. For input *x*_*t*_, it takes *x*_*t*_ and hidden state *h*_*t*−1_ from the previous time step *t* − 1. It computes a new hidden state *h*_*t*_ and is again passed for the next time step. These operations of GRU are governed by the following functions:

$$\begin{array}{cc} & z_{t}=\sigma \left(W_{z}x_{t}+W_{hz}h_{t-1}+b_{z}\right),\\ & r_{t}=\sigma \left(W_{r}x_{t}+W_{hr}h_{t-1}+b_{r}\right),\\ & g_{t}=\text{tanh}\left(W_{g}x_{t}+W_{hg}\left(r_{t}⊗h_{t-1}\right)+b_{g}\right),\\ & h_{t}=z_{t}⊗h_{t-1}+\left(1-z_{t}\right)⊗g_{t} \end{array}$$

where *σ* and tanh represent the sigmoid and hyperbolic tangent activation functions respectively. The operator ⊗ is the element-wise product, *W*, *W*_*h*_ are the weight matrices, and *b* are bias vectors.

### 4.3 Model assessment matrices

The prediction accuracy and the reliability of the models is assessed using root mean squared error (RMSE), Mean absolute percentage error (MAPE), and the linear correlation coefficient (R). A ML model with the smallest RMSE and MAPE along with the greatest R would be considered as the best model. The analytical structure of these metrics are given below:

$$\begin{array}{c} RMSE=\sqrt{\frac{1}{N}\sum_{i=1}^{N}{\left(y_{i}-\hat{y_{i}}\right)}^{2}}, \end{array}$$ (2)

$$\begin{array}{c} MAPE=\frac{1}{N}\sum_{i=1}^{N}|\frac{y_{i}-\hat{y_{i}}}{y_{i}}|, \end{array}$$ (3)

$$\begin{array}{c} R=\frac{\sum_{i=1}^{N}\left(y_{i}-\bar{y_{i}}\right)\left(\hat{y_{i}}-\bar{\hat{y_{i}}}\right)}{\sqrt{\sum_{i=1}^{N}(y_{i}-\bar{y_{i}}{)}^{2}(\hat{y_{i}}-\bar{\hat{y_{i}}}}{)}^{2}} \end{array}$$ (4)

where,

*y*_*i*_: Original closing prices,

$\bar{y_{i}}$ : The mean of the original closing prices,

$\hat{y_{i}}$ : Predicted closing prices,

$\bar{\hat{y_{i}}}$ : The mean of the predicted closing prices,
*N*: Number of observations.

## 5 Experiment setting and results

### 5.1 Experimental setup

The primary objective of this research is to see the effect of financial news on stock prediction. To examine the effect of financial sentiments on stock price predictions, we used two datasets—(I) Fundamental data and (II) Combined data. Fundamental data consist of only stock market data for the S&P 500 index, whereas combined data is fundamental data with a corresponding financial news sentiment score. Each of these datasets is divided into three subsets: training, validation, and testing. The overall distribution for each subset’s time range and corresponding samples are listed in Table 5. The training set contains the data from June 9, 2008, to July 1, 2017, while the test set ranges from August 3, 2020, to November 5, 2021. From the training set, 25% of the data is separated for validation.

**Table 5:** Overall distribution of training, validation, and test data.

Data	Dates(mm/dd/yyyy)	No of samples
Complete	06/09/2008 to 07/01/2016	2333
	08/03/2020 to 11/05/2021	
Training	06/09/2008 to 06/26/2014	1518
Validation	06/27/2014 to 07/01/2017	502
Test	08/03/2020 to 11/05/2021	313

As our goal is to perform the comparative analysis of the outcome of LSTM and GRU model architecture using two different data sets under identical conditions. Thus, we develop the four models —two using fundamental data (LSTM, GRU) and the remaining two using combined data (LSTM-News, GRU-News). The normalized three-dimensional training and validation dataset is fed to LSTM and GRU models. The models are trained in a supervised learning environment with the mean square error as the loss function. During the training process, we fixed the time step to 5. Both models (LSTM and GRU) are initialized with the input layers, followed by two hidden LSTM layers (two hidden GRU layers for GRU), and a dense layer with linear activation function, respectively. The hyperparameters: number of neurons in each layer, batch size, optimizer, number of epochs, and learning rate were optimized. The optimal set of hyperparameters for each model architecture is presented in Table 6.

**Table 6:** List of best hyperparameters for the models.

Models	No of Neurons		Optimizer	Learning rate	Batch size	No of epochs
	1 hidden layer	2 hidden layer				
LSTM-News & LSTM	100	50	Adam	0.001	8	30
GRU-News & GRU	100	50	Adam	0.001	8	30

### 5.2 Experimental results

We ultimately compare the performance of the ML models with news scores (LSTM-News and GRU-News) against those without news scores (LSTM and GRU) models utilizing the test data via the performance matrices presented in Table 7. The correlation coefficient (R) does not vary significantly between the models and shows virtually a perfect correlation. However, based on the lowest RMSE and MAPE, GRU-News performs best among the four models. We can conjecture a ranking of our models as GRU-News, LSTM-News, LSTM, and GRU, respectively with an order of decreasing performance metrics.

**Table 7:** Model performance metrics of four models obtained using test datasets.

Model	LSTM	GRU	LSTM-News	GRU-News
RMSE	39.9628	41.7668	34.8554	34.7262
R	0.9960	0.9961	0.9962	0.9962
MAPE	0.8267	0.8629	0.6703	0.6687

**Fig 5:**
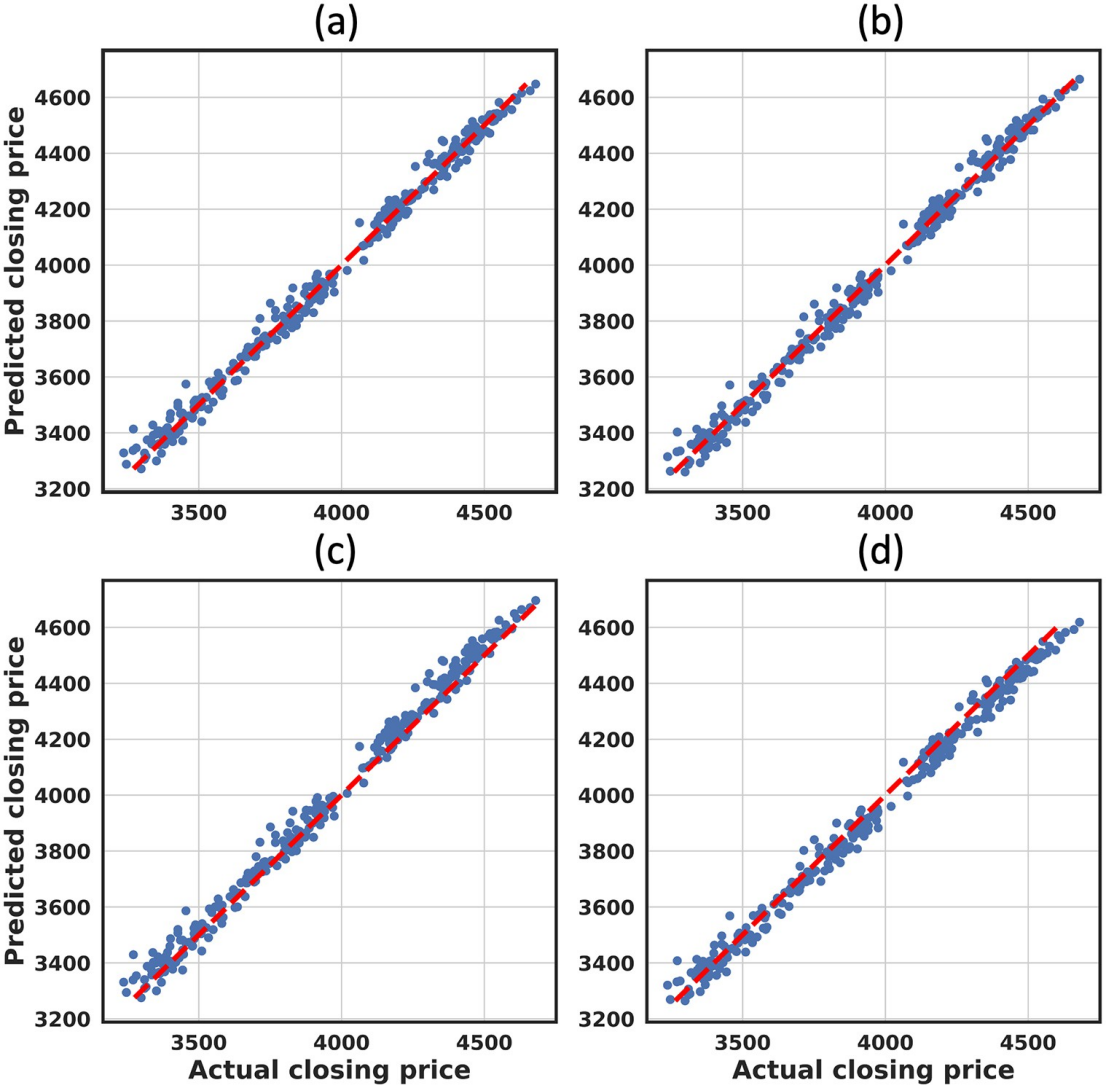
Scatter plots between the actual closing price and predicted closing price of test data corresponding to the models: (a) LSTM-News, (b) GRU-News, (c) LSTM, and (d) GRU. The best-fit linear equation (y = x) is represented by the red dotted line.

The scatter plot of the true values versus the predicted values of closing price for test data are plotted in (Fig 5(a)–5(d)). These plots provides useful information to gauge the goodness of fit of the model. If the predicted values are close to the actual values, the plot resembles a straight line at a 45-degree angle with the horizon, resulting in *R* close to 1.

**Fig 6:**
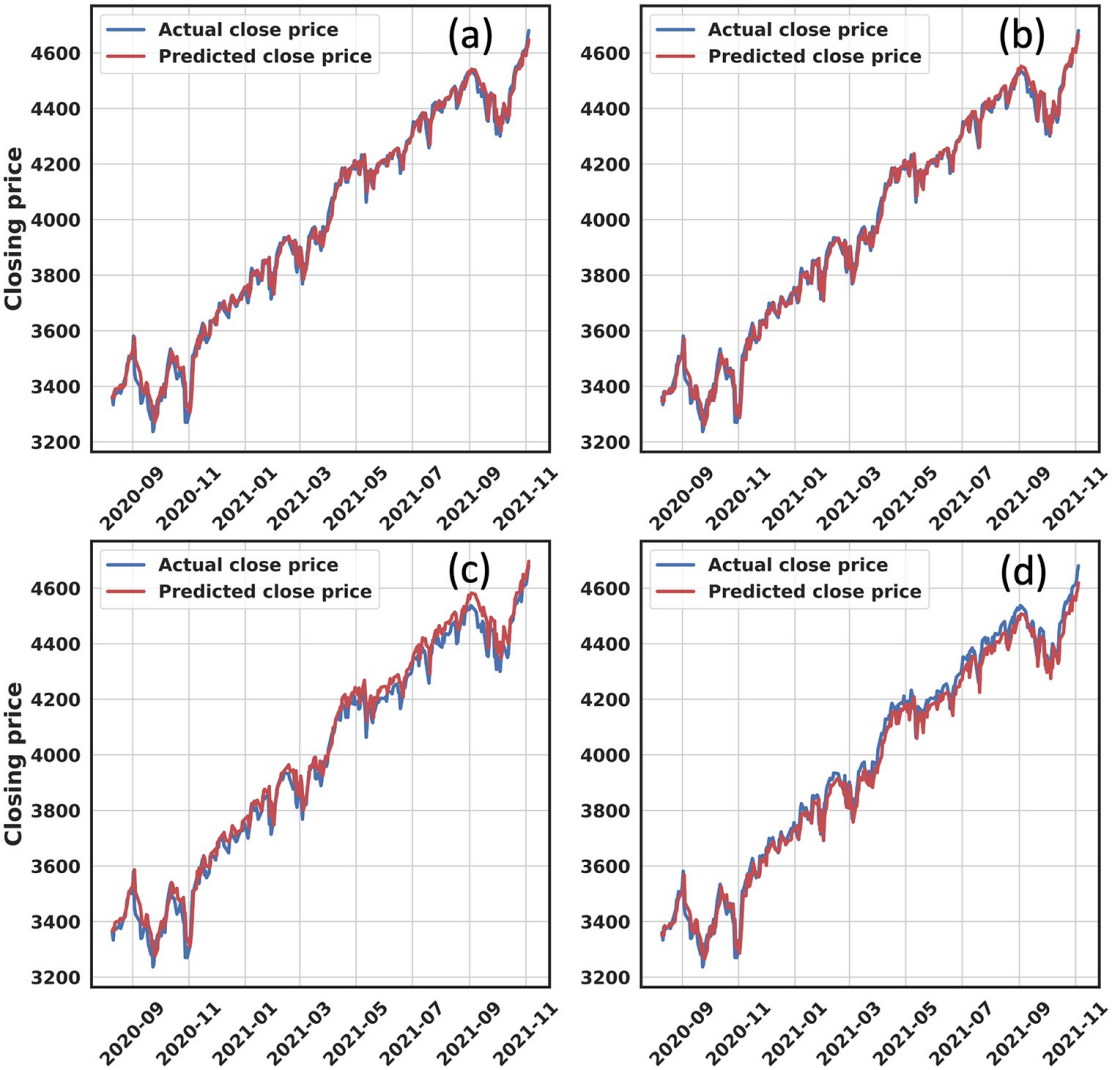
Time series plots between the actual closing price and predicted closing price of test data corresponding to the models: (a) LSTM-News, (b) GRU-News, (c) LSTM, and (d) GRU.

The time series plots presented in (Fig 6(a)–6(d)), show the pattern of actual closing price to the predicted closing price of the employed model architecture. The predicted closing price nearly overlaps with the actual closing price in all cases. It further verifies the fact that all four models sufficiently capture the trend of closing price despite of having various irregularities. In a nutshell, Figs 5 and 6 speaks tons of information about the influence of financial news in stock price prediction in terms of model accuracy.

### 5.3 Statistical analysis

The model assessment matrices and visualization techniques discussed above indicate the model that incorporates the financial news data better represents the behavior of the stock market. We would further like to validate the fact that the “performance of each model is different or not” using statistical analysis. It can be executed from both parametric and non-parametric tests.

One-way analysis of variance (ANOVA) is considered as the first method under the umbrella of the parametric test. Even though it is easy to implement and interpret, it may not predict the p-value accurately if the data are not normally distributed [67]. Therefore, before implementing the one-way ANOVA, the condition of normality must be satisfied.

**Fig 7:**
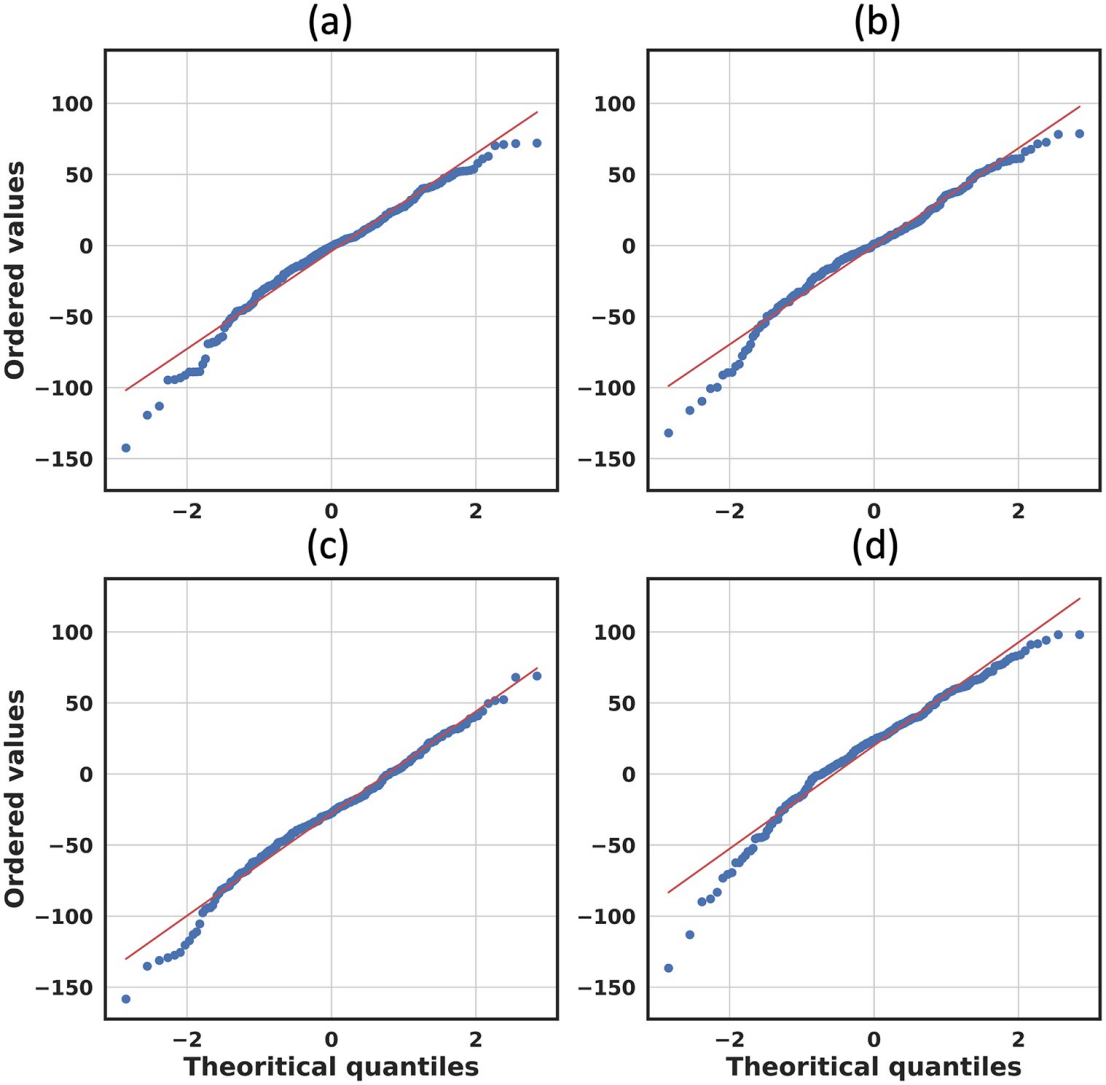
Normal QQ-plots of errors (actual closing price—predicted closing price) of test data corresponding to the models: (a) LSTM-News, (b) GRU-News, (c) LSTM, and (d) GRU. The quantile of normal distribution and errors are represented by a solid red line and blue dots, respectively.

Quantile quantile (QQ) plot [68] has been employed to test the normality in the data. The error (true closing price—predicted closing price) of QQ plots of the test data (Fig 7(a)–7(d)) provide initial insights on normality. Technically speaking, the quantile of normal distribution (straight line in Fig 7) and the quantile of errors (blue dots in Fig 7) should overlap if the data is normally distributed. (Fig 7) reveals the fact that errors are not normally distributed. Therefore, the situation demands hypothesis tests to draw the conclusions about non-normality of the errors.

The hypothesis test for the normality of each error and corresponding p-values are listed in (Table 8) using the method described by D’Agostino [69]. Since the p-values are very small (Table 8), we reject the null hypothesis (errors are normally distributed). Therefore, there is sufficient evidence to conclude the fact that the error obtained from four models—LSTM, GRU, LSTM-News, and GRU-News —are far away from the normal distribution. Statistically speaking the populations corresponding to these errors are not normally distributed. These results conclude the assumptions for a parametric test are not applicable in the given scenario.

**Table 8:** Hypothesis together with p-values of the normality test.

Hypothesis	p-value
Are the LSTM-News errors normally distributed?	30.1166 × 10^−7^
Are the GRU-News errors normally distributed?	23.90221 × 10^−6^
Are the LSTM errors normally distributed?	6.54102 × 10^−8^
Are the GRU errors normally distributed?	4.0378 × 10^−9^

As the non parametric test does not require to satisfy any assumptions, Kruskal Wallis test [70] is implemented first under this category. The Kruskal Wallis test is proposed to answer the following hypothesized statements:

*H*_0_: The prediction accuracy of the models are not significantly different.

*H*_1_: At least one model has significantly different prediction accuracy than others.

Kruskal Wallis test statistic and the p-value of the test are 123.1135 and 1.6475 × 10^−26^ respectively. Since the p-value of the test is approximately equivalent to zero, so we reject the null hypothesis. In other words, there is sufficient evidence to conclude that at least one model has significantly different prediction accuracy than the others.

However, the Kruskal Wallis test does not identify the number of models that have different prediction accuracy. To identify the models with different prediction accuracy, we perform a pairwise comparison of the models using the Mann-Whitney test [71]. The p-values of the Mann-Whitney test are presented in Table 9.

**Table 9:** P-values of the Mann-Whitney test for pairwise comparisons within the models.

	LSTM-News	LSTM	GRU-News	GRU
LSTM-News		5.98 × 10^−15^	0.2778	2.08 × 10^−18^
LSTM			1.28 × 10^−11^	0.2261
GRU-News				9.25 × 10^−15^

Looking at Table 9, we can draw the final conclusion that the performance of the LSTM-News and LSTM is significantly different. Moreover, LSTM-News has better prediction accuracy than LSTM. Similarly, GRU-News shows better prediction accuracy than GRU. In either of the cases, the p-value presented in Table 9 and performance metrics (Table 7) is considered as primary evidence to end up with the conclusion. Moreover, we can further conclude the following additional hypothesis:

- The prediction accuracy of LSTM-News is significantly better than the GRU.
- The prediction accuracy of GRU-News is significantly better than the LSTM.
- There is not sufficient evidence to conclude that the performance of LSTM and GRU are significantly different, though LSTM has slightly better performance metrics than GRU.
- There is not sufficient evidence to conclude that the performance of LSTM-News and GRU-News are significantly different, though GRU-News has slightly better performance metrics than LSTM-News.

### 5.4 Ethics and implications

The study uses publicly available fundamental stock market data and web-scraped financial news data without manipulation. A series of statistical evidence further supports the reported performance of the model to make the result trustable to the model’s reliability and robustness. One can use the results as additional information to boost confidence in stock investment decisions. The investment decision should not rely solely on this research outcome. Investors are suggested to use their experience and risk tolerance behavior and consider other lurking variables based on the existing market situations. Thus, one can benefit if the current market conditions are appropriately analyzed and amalgamated with the model’s outcome. This research shows the promising possibility of using GRU and CNN architecture utilizing financial news data in combination with fundamental stock data to delineate the cone of uncertainty in stock price prediction.

## 6 Conclusions

Stock price prediction is gaining popularity for all the stakeholders involved directly/indirectly in making a responsible financial decision. Precise and consistent prediction is challenging due to its volatile, nonlinear, noisy, chaotic, and fuzzy behavior. It is a rational idea to identify whether structured numerical data or unstructured text data or a combination of both, influence stock price prediction. The current literature lacks a comparative analysis of stock price prediction with or without incorporating unstructured data that focuses on human sentiment. The primary objective of this article is to conduct a comparative study of LSTM and GRU under identical conditions by utilizing multifaceted information and to identify the influence of text data in stock price prediction. A comprehensive data-driven approach via hyperparameter tuning reveals that including financial data produces better LSTM and GRU architecture performance. Furthermore, stock price prediction can significantly improve when the stock market’s fundamental data is amalgamated with financial news data. Thus, the employed model outcome quantitatively supports the longstanding belief in sentiment and social media influence on the stock price. Our result further endorsed the standard assessment metrics (RMSE, MAPE, and R) and the series of statistical tests to validate its reliability and robustness.
